# Secular trends in smoking during pregnancy according to income and ethnic group: four population-based perinatal surveys in a Brazilian city

**DOI:** 10.1136/bmjopen-2015-010127

**Published:** 2016-02-01

**Authors:** Mariangela F Silveira, Alicia Matijasevich, Ana Maria B Menezes, Bernardo L Horta, Ina S Santos, Aluisio J D Barros, Fernando C Barros, Cesar G Victora

**Affiliations:** 1 Faculty of Medicine, Maternal and Child Department and Post Graduation Program in Epidemiology, Federal University of Pelotas, Pelotas, Brazil; 2 Department of Preventive Medicine, School of Medicine, University of São Paulo Pelotas, Pelotas, Brazil; 3 Faculty of Medicine, Post-Graduate Program in Epidemiology, Federal University of Pelotas, Pelotas, Brazil; 4 Post-Graduate Program in Health and Behavior, School of Medicine, Catholic University of Pelotas, Pelotas, Brazil

**Keywords:** smoking, inequalities, pregancy, trends

## Abstract

**Objectives:**

To assess socioeconomic and ethnic inequalities in smoking during pregnancy over three decades (1982–2011).

**Setting:**

Population-based study in Pelotas City, Brazil.

**Participants:**

All urban women giving birth in the city hospitals in 1982 (5909), 1993 (5223) and 2004 (4201), plus all urban and rural women delivering from January 2011 to April 2012 (6275).

**Primary outcome:**

Self-reported smoking during pregnancy.

**Results:**

The prevalence of smoking during pregnancy fell from 35.7% in 1982 to 21.0% in 2011. In each survey, prevalence decreased with increasing income (p<0.001). In the poorest quintile, smoking fell by 27.4% in the period studied compared to 67.1% in the wealthiest quintile. In all surveys, prevalence was lower among white women than among those who classified themselves as black or brown (p<0.001). Over time, smoking declined by 50.0% among the former and 30.7% among the latter. Absolute and relative inequalities both increased over time.

**Conclusions:**

The reduction in smoking during pregnancy was primarily due to a decline among white, high-income women. Further efforts are needed to reduce smoking among all population groups.

**Strengths and limitations of this study**

- The study's main strength is the analysis of four population-based studies in the same city over a 30-year period, using highly comparable methodology, with very high response rates.
- Long-term analyses of socioeconomic inequalities in smoking in low-income and middle-income countries are rare in the literature.
- The study population's marked social stratification provides an excellent opportunity for assessing inequalities in smoking prevalence among socioeconomic and ethnic groups.
- A limitation is that data originate from a single city in Southern Brazil, rather than from the whole country.
- Another limitation is that information on smoking during pregnancy was self-reported at the time of childbirth, as was information on family income.

## Introduction

Smoking during pregnancy leads to harmful effects on the fetus, including intrauterine growth restriction^1^ and preterm delivery.^2^ Globally, tobacco smoking prevalence has been declining, but there are important variations by country.^3^ Smoking prevalence among pregnant women^4^ and among women of reproductive age^5^ also differs markedly among low-income and middle-income countries. The socioeconomic patterning of smoking also varies from country to country. For example, while in Mexico and Turkey smoking is more prevalent among more educated women, in Uruguay and the Philippines the trend is in the opposite direction.^5^

In terms of time trends, studies from several high-income countries show that the decline in smoking during pregnancy varies according to the woman's education and socioeconomic position. This has been observed in Canada,^6^ Norway,^7^ the Netherlands,^8^ France,^9^ Spain,^10^ Australia^10^ and Sweden.^11^ Several of these studies report that absolute reductions in smoking prevalence—expressed as a difference in per cent points—tend to be larger among the poorer and less-educated women than among richer, more educated women. This is because the latter had lower prevalence at baseline and therefore there was less scope for absolute reductions. In contrast, relative reductions expressed as a per cent of the baseline prevalence are typically faster among the better off. This combination of decreased absolute inequalities combined with increased relative inequalities is not uncommon in studies of equity trends.^12^

Maternal ethnicity has also been found to be associated with smoking during pregnancy. In North America, black women appear to be less likely to smoke than white women,^13–15^ although some studies report similar rates.^16^ In Brazil, this pattern is reversed in most^17–19^ but not all^20^ studies. A study from the USA reports that, from 2000 to 2005, rates of smoking during pregnancy remained stable for white and black women,^21^ although in the 25–34-year age group there was an increase for blacks.

In contrast to the ample literature from high-income countries, we were unable to find any reports on disaggregated time trends from low-income or middle-income countries. The availability of four studies carried out between 1982 and 2011, each covering all births in the same Brazilian city, allowed us to investigate how social and ethnic group inequalities in smoking during pregnancy are evolving over time in a middle-income setting.

## Methods

Four population-based studies were carried out in 1982, 1993, 2004 and 2011, in the city of Pelotas, in Southern Brazil. The city's population increased from 230 000 in 1982 to 340 000 at present.

The first three studies were the perinatal interviews of birth cohorts, each including all births occurring in all the city's maternity hospitals during each year.^22–24^ Over 98% of the city births took place in these hospitals. Mothers who were resident in the Pelotas urban area were interviewed soon after birth on biological, demographic, reproductive, behavioural and socioeconomic characteristics. The fourth study was the Newborn Cross Sectional Study, a screening component of the Intergrowth 21st multicentre study. From January 2011 to April 2012, all mothers giving birth in the city's maternity hospitals were interviewed. Unlike the previous cohorts, this study did not exclude mothers from the rural areas and neighbouring towns, who account for 13% of all births. All women were included in the present analyses, but only a subset of those who complied with strict criteria were included in the Intergrowth 21st standards.^25^

In the four studies, similar questions were used to obtain information on smoking, income and skin colour. Smoking during pregnancy was self-reported and defined as consumption of at least one cigarette a day, during at least part of the gestation. Information on total family income (including wages and other monetary earnings, such as pensions and benefits) during the previous month was collected at the time of delivery and the women were divided into quintiles. Using the standard classification for ethnicity adopted by the Brazilian Census Bureau (Brazil; IBGE; Atlas do Censo Demográfico de 2010; Rio de Janeiro, IBGE, 2010), mothers classified their own skin colour as black, brown or white. A recent analysis comparing self-reported skin colour with genomic ancestry markers, carried out in three Brazilian samples including the city of Pelotas, showed a high degree of consistency.^26^

For linear trend tests, χ^2^ was used to compare prevalence of smoking by income and skin colour. To summarise income-related inequalities, we calculated four indicators.^27^ Two of these were based on simple comparisons of the poorest (Q1) and richest quintiles (Q5): the difference and ratio between the corresponding prevalence of smoking. We also calculated two indicators of inequality that take the whole distribution of income into account, instead of only the extreme groups. The first is the slope index of inequality (SII) expresses the absolute difference in the outcome, in percentage points, between the extremes of the income distribution, based on a logistic regression model.^28^ The second is the concentration index (CIX),^29^ which is a similar concept to the Gini index for income distribution. CIX is expressed in a scale from −100 to +100, with full equality being equal to zero. For smoking during pregnancy, both summary measures tend to be negative because the rich usually have lower prevalence than the poor. The Q1–Q5 difference and the SII express absolute inequality, whereas Q1/Q5 ratio and the CIX express relative inequalities.^27^

The four studies were approved by the Medical Ethics Committee of the Federal University of Pelotas, affiliated with the Brazilian National Commission for Research Ethics (CONEP).

## Results

The total numbers of single births in the four studies were 5909, 5223, 4201 and 6275.

Refusal rates were 1% in all studies. Missing values for income, skin colour and smoking prevalence were all below 2%, and these observations were excluded from the analyses.

Table 1 shows sociodemographic characteristics of the women in the four studies. There were positive trends in education. More mothers classified themselves as black or brown in the more recent cohorts than in the past. There was a proportionate increase in mothers aged 35 years or older, in those who do not live with a partner, and—to a lesser extent—in primiparae.

**Table 1:** Maternal characteristic in the four studies, 1982–2011

Variables	Pelotas 1982 n (%)	Pelotas 1993 n (%)	Pelotas 2004 n (%)	Intergrowth 2011 n (%)	p Value*
Ever smoked during pregnancy (†)					<0.001
No	3802 (64.3)	3486 (66.7)	3040 (72.4)	4954 (79.0)	
Yes	2107 (35.7)	1737 (33.3)	1161 (27.6)	1318 (21.0)	
Maternal education					
0	317 (5.5)	131 (2.5)	44 (1.1)	25 (0.4)	<0.001
1–4	1605 (27.6)	1334 (25.6)	606 (14.6)	504 (8.0)	<0.001
5–8	2425 (41.7)	2416 (46.3)	1719 (41.3)	2366 (37.7)	<0.001
≥9	1462 (25.2)	1335 (25.6)	1791 (43.0)	3378 (53.9)	<0.001
Family income (quintiles)					
1st (poorest)	1159 (19.9)	1054 (20.2)	862 (20.5)	1229 (19.6)	0.783
2nd	1166 (20.0)	1170 (22.4)	855 (20.3)	1381 (22.0)	0.079
3rd	1166 (20.1)	931 (17.8)	815 (19.4)	1247 (19.9)	0.779
4th	1162 (20.0)	1042 (20.0)	851 (20.3)	1257 (20.0)	0.849
5th (wealthiest)	1163 (20.0)	1026 (19.6)	818 (19.5)	1161 (18.5)	0.044
Skin colour					<0.001
White	4845 (82.0)	4031 (77.2)	3062 (72.9)	4232 (67.5)	
Brown/black	1061 (18.0)	1190 (22.8)	1139 (27.1)	2035 (32.5)	
Age (years)					
<20	908 (15.6)	918 (17.6)	803 (19.1)	1076 (17.2)	0.004
20–34	4339 (74.6)	3725 (71.3)	2829 (67.4)	4351 (69.3)	<0.001
≥35	568 (9.8)	579 (11.1)	567 (13.5)	847 (13.5)	<0.001
Marital status					<0.001
With partner	5419 (91.8)	4578 (87.6)	3507 (83.5)	5428 (86.5)	
Single mother	485 (8.2)	645 (12.4)	694 (16.5)	846 (13.5)	
Parity					
0	2299 (39.6)	1826 (35.3)	1662 (39.6)	2703 (43.1)	<0.001
1	1642 (28.2)	1429 (27.7)	1092 (26.0)	1789 (28.5)	0.741
≥2	1873 (32.2)	1913 (37.0)	1446 (34.4)	1782 (28.4)	<0.001

*χ^2^ Test for trend over time.

†Missing information for 3 women in the Intergrowth study.

Maternal characteristics are broken down by income quintile in online webtable 1. Low-income mothers tended to have lower education, were less likely to classify themselves as white and as having a partner, were younger and had higher parity.

We report on socioeconomic inequalities in terms of income because this variable can be divided into quintiles and is easily amenable to studying trends in equal-sized population groups. This is not the case for education, where the size of the groups varied markedly over time (see table 1). Information on age and parity was used to describe the population, but we chose not to treat these variables as confounders of the association between income and smoking, as both age and pattern are socially determined (and therefore do not comply with the criteria for characterising confounding variables).

**Figure 1:**
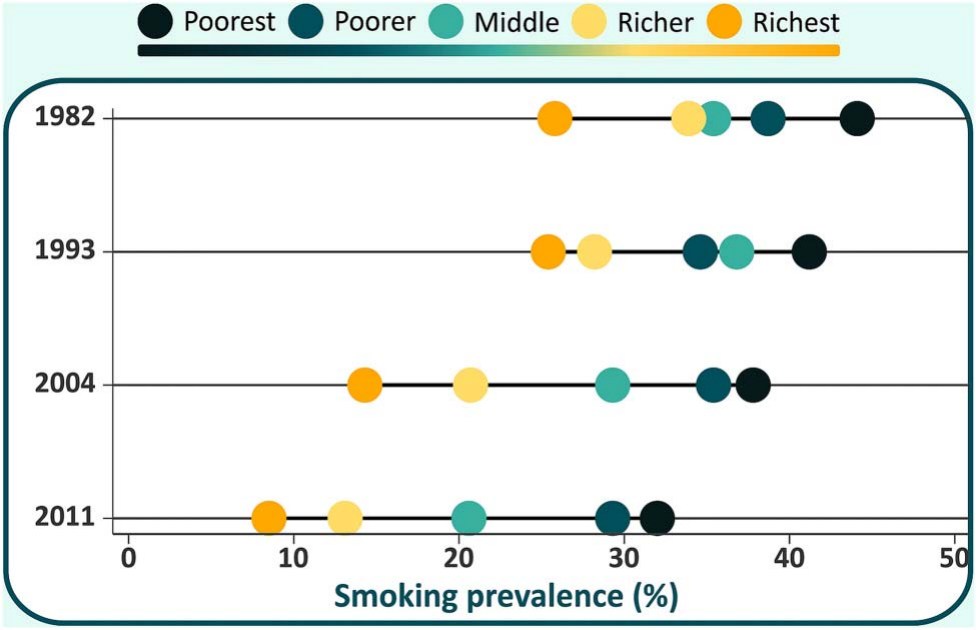
Smoking prevalence by family income quintile in the four studies, 1982–2011.

Figure 1 and table 2 show smoking prevalence by income quintile in the four studies. Overall smoking prevalence declined by 41% from 1982 to 2011 (table 2). Within each cohort, smoking was significantly (p<0.001) and inversely related to family income. Declines were observed in all income groups, but were considerably higher among the richest women (67%) compared to the poorest (27%). The 1.7-fold ratio between poorest and richest that was observed in 1982 increased to 3.8 in 2011. The test for interaction between year and income was statistically significant (p<0.001).

**Table 2:** Smoking prevalence according to income and summary measures of inequality in the four studies

Income quintile	Year				Reduction	Average annual absolute reduction
	1982 (%)	1993 (%)	2004 (%)	2011 (%)	1982–2011 (%)	Percentage points per year
Poorest	44.1	41.2	37.8	32.0	27.4	−0.39
2nd	38.7	34.6	35.4	29.3	24.3	−0.27
3rd	35.4	36.8	29.3	20.6	41.8	−0.51
4th	33.9	28.2	20.7	13.1	61.4	−0.70
Richest	25.8	25.4	14.3	8.5	67.1	−0.63
Whole sample	35.7	33.3	27.6	21.0	41.0	−0.50
Summary measures						
Slope index	−20.7	−19.0	−30.85	−31.6		
Concentration index	−0.09	−0.087	−0.176	−0.244		
Poorest-richest difference	18.3	15.8	23.5	23.5		
Poorest/richest ratio	1.7	1.6	2.6	3.8		

All p levels for time trends <0.001.

The SII uses information from the five quintiles to express the absolute difference between the extremes of the income scale. In 1982, this difference was −20.7% points. The respective values for 1993, 2004 and 2011 were −19.0, −30.85 and −31.6 points. The CIX, which reflects relative inequalities, changed from −0.09 in 1982 and 1993, to −0.18 in 2004 and −0.24 in 2011. Negative values of this index indicate that the poor have higher smoking prevalence than the rich.

**Figure 2:**
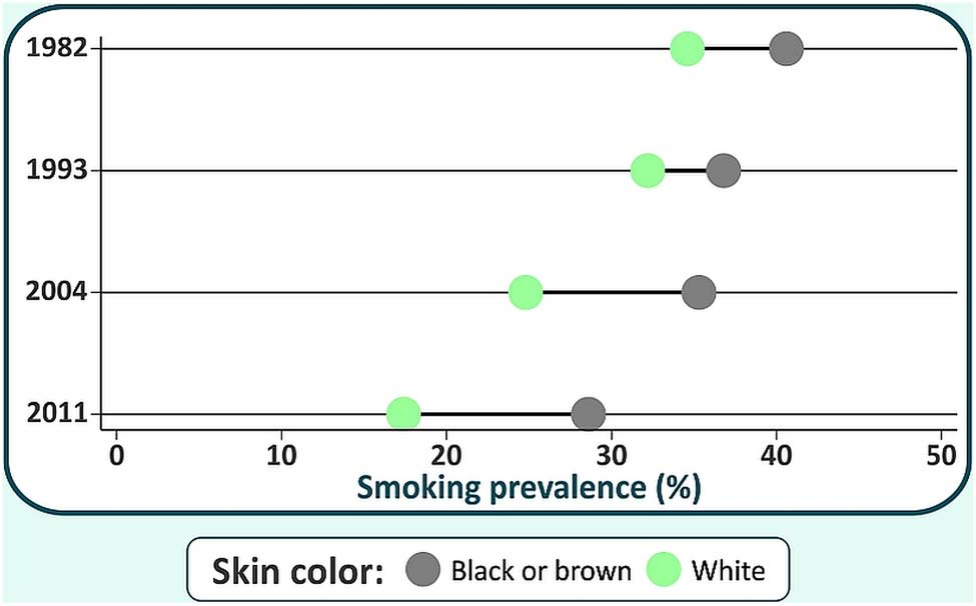
Smoking prevalence by skin colour in the four studies, 1982–2011.

Figure 2 shows the results for skin colour. Smoking prevalence for white mothers was 34.6%, 32.2%, 24.8% and 17.4% in the four studies, whereas the respective values for black or brown mothers were 40.6%, 36.8%, 35.3% and 28.6% (figure 2). Therefore, smoking declined by 17.2% points, or 50% of the original 1982 prevalence, among white women, compared to 12.0% points, or 30.7% of the 1982 level, among black or brown women. Based on the above data, absolute inequalities between the two groups increased from 6.0% to 11.2% points over time, and relative inequalities, from a prevalence ratio of 1.2 to 1.6.

## Discussion

To our knowledge, this is the first published analysis of long-term trends in smoking during pregnancy in a middle-income setting. The comparison was made possible by the existence of four population-based studies in the same Brazilian city that used comparable methods to assess smoking prevalence, socioeconomic position and ethnicity.

Our study's limitations include the fact that smoking habits and family income were both based on the women's report at the time of childbirth. Laboratory measures of exposure to cigarette smoke were not available, and even if they were, they would not reflect smoking at any time during pregnancy. The possibility of differential reporting by income groups cannot be ruled out, but it should be pointed out that national surveys in Brazil^30^ and other low-income and middle-income countries^31^ also show similar socioeconomic gradients.

A four-stage model for smoking prevalence and tobacco-related mortality in high-income countries was proposed in 1994, by Lopez *et al*.^32^ The model predicted that smoking prevalence increased first among men, followed by an increase among women. The decline among men started to occur while prevalence was still increasing among women. Tobacco-related deaths would show similar patterns, with an offset of three to four decades, as mortality is associated with previous, rather than current smoking. An assessment of the model in 2012^33^ showed that it provided a reasonable explanation for recent trends in high-income countries, but that ‘its relevance to developing countries could be improved by describing the stages of the epidemic separately for men and women’. Data on long-term time trends in smoking prevalence from middle-income countries are scarce, and our results confirm earlier reports from Brazil,^34^ ^35^ showing that women have already reached the declining section of the smoking curve.

Even fewer data are available on long-term trends in smoking by socioeconomic status outside industrialised countries, where the concentration of smoking among the poor had already been observed decades ago.^36^ The inverse equity hypothesis^37^ predicts that new preventive behaviours are first adopted by the better off groups in the population, who have greater access to information, education and economic resources for prevention. As a consequence, inequalities tend to increase in the short term. This seems to be the case for smoking patterns among our pregnant women, which declined markedly faster among the rich than the poor, resulting in a wider gap in 2011 than was present 30 years earlier. The gap increased in absolute terms, from a difference of 18.3 percentage points in smoking prevalence between the poorest and richest quintiles in 1982 to 23.5 percentage points in 2011. It also increased in relative terms, with the prevalence ratio between the poorest and richest quintiles increasing from 1.7 to 3.8 times.

The inverse equity hypothesis also predicts that, when prevalence among the better off becomes very low, absolute inequalities will likely fall as there is less additional scope for improvement among the rich than among the poor. In Pelotas, it seems that this is yet to happen, as prevalence in the rich was still 8.5% in 2011.

In contrast, studies from high-income countries reviewed in the introduction show that prevalence among the rich has already reached very low levels, so that absolute inequalities are being reduced because of continued—albeit often slow—progress among the poor. In Sweden, for example, between 1982 and 2001, the absolute gap between educational groups fell from 14.5% to 10.2% points, whereas the corresponding ORs increased from 5.6 to 14.2, indicating increased relative inequalities.

Our results on ethnic group inequalities also show an increasing gap over time both in absolute (from 6.3% to 11.1% point difference between brown or black women compared with whites) and in relative terms (from a ratio of 1.2 to 1.6). Data from the USA for 2000–2005 do not show a differential trend according to skin colour,^21^ but it is possible that studies with longer time spans might reveal significant trends.

Our results show that there have been declines in smoking prevalence in all social groups, but particularly among the better off, leading to exacerbated socioeconomic inequalities. A similar trend was observed for ethnicity, with a widening gap between white and black or brown women. Although smoking prevalence in all groups is still unacceptably high, focusing on women of low socioeconomic position and on black and brown women will likely contribute to a faster decline in this behaviour that has such important consequences for women and their children.
